# Cellular Apoptosis of Hemocytes from *Dendrolimus* *tabulaeformis* Tsai et Liu Larvae Induced with the Secondary Metabolites of *Beauveria* *brongniartii* (Sacc.) Petch

**DOI:** 10.1371/journal.pone.0071600

**Published:** 2013-08-07

**Authors:** Jinhua Fan, Yingping Xie, Jiaoliang Xue, Yingling Zhang, Qian Yang

**Affiliations:** School of Life Science, Shanxi University, Taiyuan, China; Fundación Investigación Sanitaria Illes Balears, Spain

## Abstract

To investigate the effect of the secondary metabolites of entomopathogenic fungus on the hemocyte immunity of host insect, the secondary metabolite complex (SMC) of *Beauveria* *brongniartii* was used in three concentrations (5.5, 55, and 550 µg/mL), and the 4^th^ instar larvae of the pine caterpillar *Dendrolimus* *tabulaeformis* were employed as host insects. The larvae were inoculated with the SMC solutions by injection in bioassays. Apoptosis of the larval hemocytes was observed using fluorescence microscopy (FM), transmission electron microscopy (TEM), and flow cytometry (FCM). The FM results showed that in the treated groups, larval hemocytes exhibited symptoms of early apoptosis at 6 h post-treatment by radiating a non-uniform kelly fluorescence and exhibited symptoms of late apoptosis at 12 h post-treatment by radiating a non-uniform orange fluorescence. Under TEM, the following ultra-structural changes associated with apoptosis of the larval hemocytes were observed in the treated groups: the nuclei were hypertrophied, slight folds were on the nuclear envelope, the chromatin became concentrated, the mitochondrial cristae disappeared or were disorderly, most cells developed blebs, and fibrillar aggregation appeared and accumulated in the cytoplasm. Apoptosis of the larval hemocytes was detected by FCM at 6 h post-treatment; the percentage of early apoptotic cells in the SMC 5.5, 55, and 550 µg/mL treatment groups were 11.93%, 13.10%, and 18.42%, respectively. Late apoptosis first occurred at 12 h post-treatment; the highest rate of apoptosis was 36.54 ± 4.37% at 24 h post-treatment in the SMC 55 µg/mL treatment group. In general, the cellular apoptosis rate was positively correlated with the SMC concentration and the time post-treatment. These results indicate that secondary metabolites of *B* *. brongniartii* are able to attack the hemocytes of *D* *. tabulaeformis* larvae and induce cellular apoptosis, thereby providing new evidence that secondary metabolites of mycopathogens can act on host immune systems.

## Introduction

Entomogenous fungi have been utilized as pathogenic agents in the biological control of pests. The infection mechanism of fungi in the host insects has been reported [1–3]. Fungal toxin plays an important role in killing insects, besides the direct infection by the fungus [4]. The toxic metabolites of entomogenous fungi include several extracellular enzymes, proteins, and low-molecular-weight compounds, including beauvericin, oosporein, and destruxin [5,6]. Studies have suggested that the potential effect of these secondary metabolites is inhibition of the immune activity of the host [7,8]. Insects have evolved an innate immunity against such invading organisms. The immune responses in the hemolymph include phagocytosis, nodulation, and encapsulation, which are usually followed by a hemocyte and humoral response [9–12]. Bandani reported that *Tolypocladium* *cylindrosporum* Gams (Deuteromycetes: Moniliaceae) and its secondary metabolites, efrapeptins, might affect the immune system of *Galleria* *mellonella* L. (Lepidoptera: Pyralidae) larvae. The total number of hemocytes in the infected insects was constant during the first 24 h and declined significantly over the following 24 h [13]. However, studies examining the effects of the secondary metabolites of entomogenous fungi on hemocyte apoptosis are scarce.

**Figure 1:**
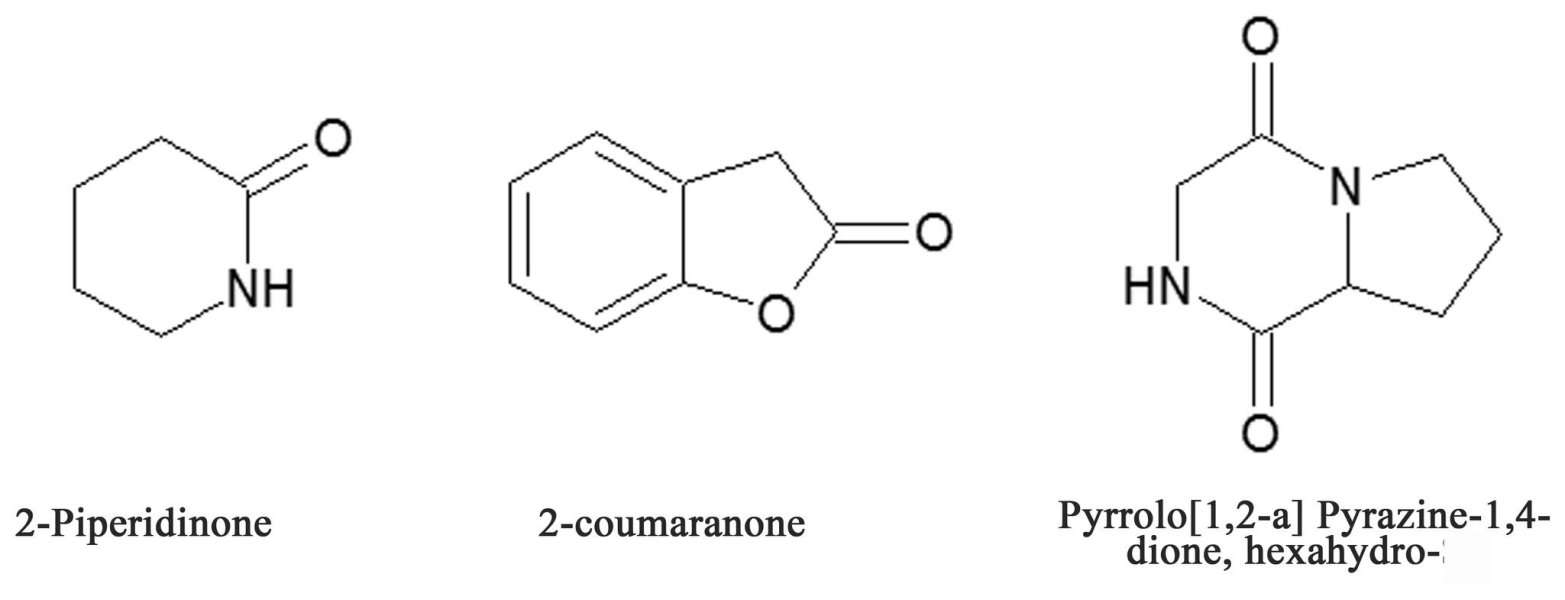
Chemical structures of the main components of the SMC of *B* *. brongniartii* strain 2382.

In the present study, the pine caterpillar, *Dendrolimus* *tabulaeformis* Tsai et Liu (Lepidoptera: Lasiocampidae), a destructive pest in the pine forests of northern China, was employed as the target host. The secondary metabolites of the entomogenous fungus *Beauveria* *brongniartii* (Sacc.) Petch (Ascomycota: Hypocreales) strain 2382 were utilized as a pathogenic toxin. We isolated this strain in 2007 from the naturally diseased cadavers of *D*. *tabulaeformis* in the pine forest in Chengde, Hebei Province, China. In our previous study, the fungal secondary metabolite was confirmed to contain 2-Piperridinone, 2-coumaranone, Pyrrolo[1,2-a]Pyrazine-1,4-dione, hexahydro (Figure 1) and certain other toxic components [14]. The objective of the present study was to investigate the effect of the secondary metabolites of the pathogenic fungus on cellular apoptosis of the hemocytes in an insect host and to obtain a new point of view to increase the understanding of how the toxic activity of the fungal secondary metabolites inhibits the immune function of hemocytes.

## Results

### Cellular apoptosis of hemocytes as measured by fluorescence microscopy (FM)

Acridine orange (AO) and propidium iodide (PI) are intercalating nucleic acid-specific fluorochromes, which emit green and red fluorescence, respectively, when they are bound to double-stranded DNA [15]. The selective permeability of the intact plasma membrane of viable cells allowed the AO, but not the PI, to enter the cell [16]; the nucleus of viable cells was stained green when observed under the fluorescence microscope. In contrast, the plasma membranes of apoptotic and necrotic cells were no longer intact, allowing the PI to enter. Comparatively, the PI produced the highest intensity emission [15]. Hence, the normal cells exhibited green fluorescence, the early apoptotic cells exhibited kelly fluorescence, and the late apoptotic cells exhibited orange fluorescence.

**Figure 2:**
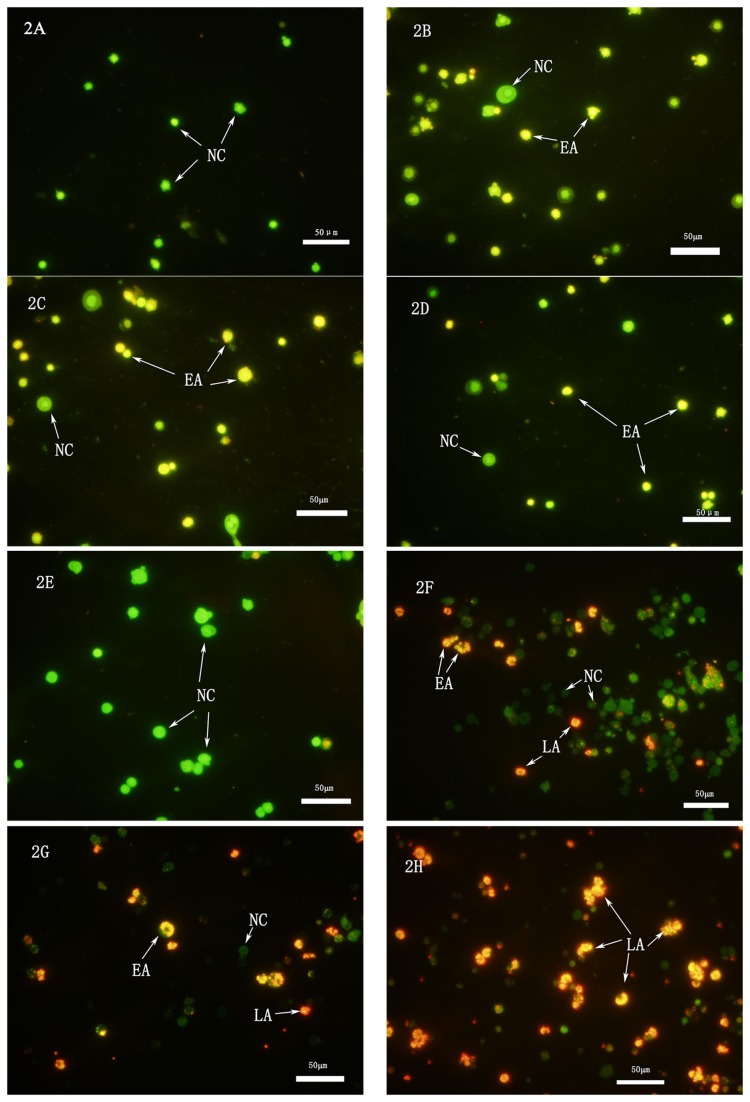
The FM photographs of hemocytes apoptosis in *D* *. tabulaeformis* larvae treated with the SMC of the fungus. 2A-2D: The hemocytes were sampled at 6 h post-treatment. The hemocytes from (2a) the control group, (2B) the 5.5 µg/mL SMC treatment group, (2C) the 55 µg/mL SMC treatment group, and (2D) the 550 µg/mL SMC treatment group. 2E-2H: The hemocytes were sampled at 12 h post-treatment. The hemocytes from (2E) the control group, (2F) the 5.5 µg/mL SMC treatment group, (2G) the 55 µg/mL SMC treatment group, and (2H) the 550 µg/mL SMC treatment group. Hemocytes with green fluorescence are normal cells, with kelly fluorescence are early apoptotic cells, and with orange fluorescence are late apoptotic cells. NC, normal hemocytes; EA, early apoptotic hemocytes; LA, late apoptotic hemocytes.

After staining with 5 µL AO/PI, the hemocytes of the *D* *. tabulaeformis* larvae were observed under the fluorescence microscope. Cellular apoptosis was distinctly recognized by the difference in fluorescence of the hemocytes. At 6 h post-treatment, hemocytes from the control group exhibited uniform green fluorescence. These hemocytes were the normal cells and their chromatin exhibited uniform distribution (Figure 2A). In the groups treated with 5.5 µg/mL of the fungal secondary metabolite complex (SMC) solution, some of the hemocytes emitted a non-uniform kelly fluorescence, the nucleus membrane were turned into blebs, indicating that these cells were in early apoptosis; chromatin aggregation also occurred in this treatment group (Figure 2B). In the 55 µg/mL SMC solution treatment groups, more hemocytes radiated non-uniform kelly fluorescence and displayed symptoms of early apoptosis (Figure 2C). Similar symptoms of early cellular apoptosis were observed in the hemocytes in the 550 µg/mL SMC solution treatment groups (Figure 2D).

At 12 h post-treatment, the hemocytes from the control groups maintained a uniform green fluorescence (Figure 2E), but the hemocytes from the SMC solution treatment groups exhibited obvious symptoms of early and late apoptosis. The early apoptotic cells emitted a non-uniform kelly fluorescence, while the late apoptotic cells emitted a non-uniform orange fluorescence and chromatin concentrated (Figure 2F–2H). With increasing concentrations of the SMC solutions in the treatment groups, the proportion of normal cells decreased, whereas the proportions of early and late apoptotic cells increased. In the groups treated with the 550 µg/mL SMC solution, majority of the hemocytes were in late apoptosis and their cellular DNA was greatly condensed, indicating that the hemocytes had been damaged by the SMC toxin (Figure 2H).

### Cellular apoptosis of hemocytes observed under transmission electron microscopy (TEM)

**Figure 3:**
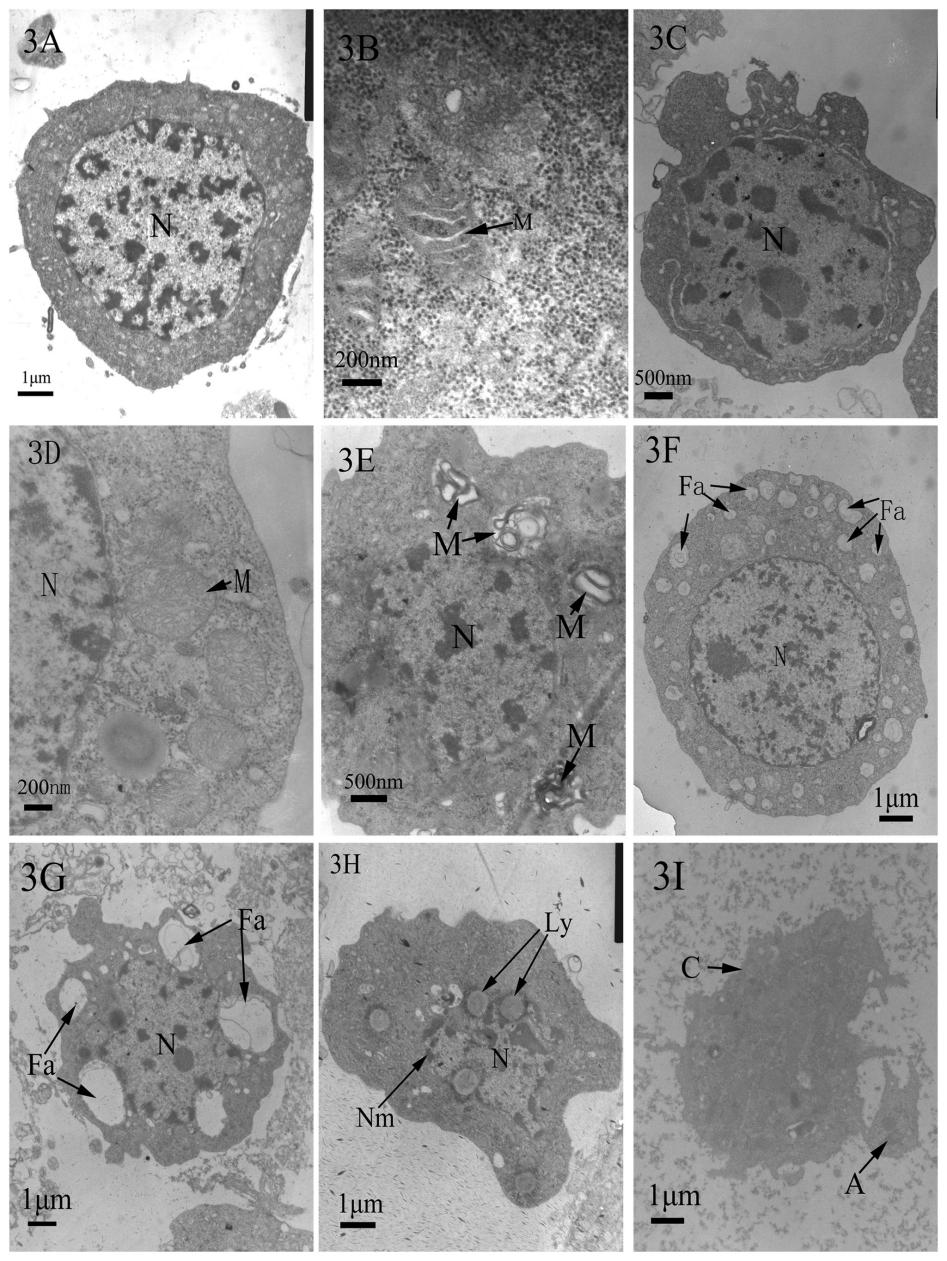
TEM micrographs of hemocytes apoptosis in *D* *. tabulaeformis* larvae treated with the SMC of the fungus. 3A and 3B: Hemocytes from the control group (3a). The normal hemocyte. N, nucleus; Bars, 1µm (3B). Mitochondria in the normal hemocyte. M, mitochondria; Bars, 200 nm. 3C-3I: Hemocytes from the SMC treatment groups at 12 h post-treatment (3C). hemocyte with hypertrophied nuclei. N, nucleus; Bars, 500 nm (3D). Mitochondria in the hemocyte. N, nucleus; M, mitochondria; Bars, 200 nm (3E). Hemocyte showing mitochondria and their lack of cristae. N, nucleus; M, mitochondria; Bars, 500 nm (3F). Hemocyte showing cytoplasmic fibrillar aggregation. N, nucleus; Fa, fibrillar aggregation; Bars, 1µm (3G). Hemocyte showing accumulated fibrillar aggregation in the cytoplasm. N, nucleus; Bars, 1 µm (3H). Hemocyte showing damaged nuclear membrane. N, nucleus; L, lysosome; Bars, 1 µm (3I). Hemocyte showing apoptotic body. A, apoptotic body; Bars, 1 µm.

Using the TEM, we observed the ultra-structural symptoms of cellular apoptosis of the hemocytes of *D* *. tabulaeformis* . At 12 h post-treatment, the hemocytes in the untreated control groups had an intact nuclear membrane, uniform chromatin (Figure 3A), an uninjured chondriosome with a smooth outer membrane, and clear and regular mitochondrial cristae (Figure 3B). Conversely, in the 55 µg/mL SMC treatment groups, the hemocytes showed the following early apoptotic characteristics: hypertrophied nuclei, a slight fold in the nuclear envelope, some chromatin concentration (Figure 3C); mitochondria gathered together and the cristae in it disorderedly ranked (Figure 3D) or missing (Figure 3E); many blebs and fibrillar aggregations in the cytoplasm (Figure 3F). At more than 12 h post-treatment with 55 µg/mL SMC, larger blebs, increased fibrillar aggregation in the cytoplasm, and deformed nuclei were observed (Figure 3G). At 24 h post-treatment, we observed that the nuclear membranes were damaged and the number of lysosome increased (Figure 3H). Finally, nuclear membranes missed in some cases and some apoptotic bodies containing cell fragmentations were observed (Figure 3I).

### Cellular apoptosis of hemocytes as measured by flow cytometry (FCM)

**Figure 4:**
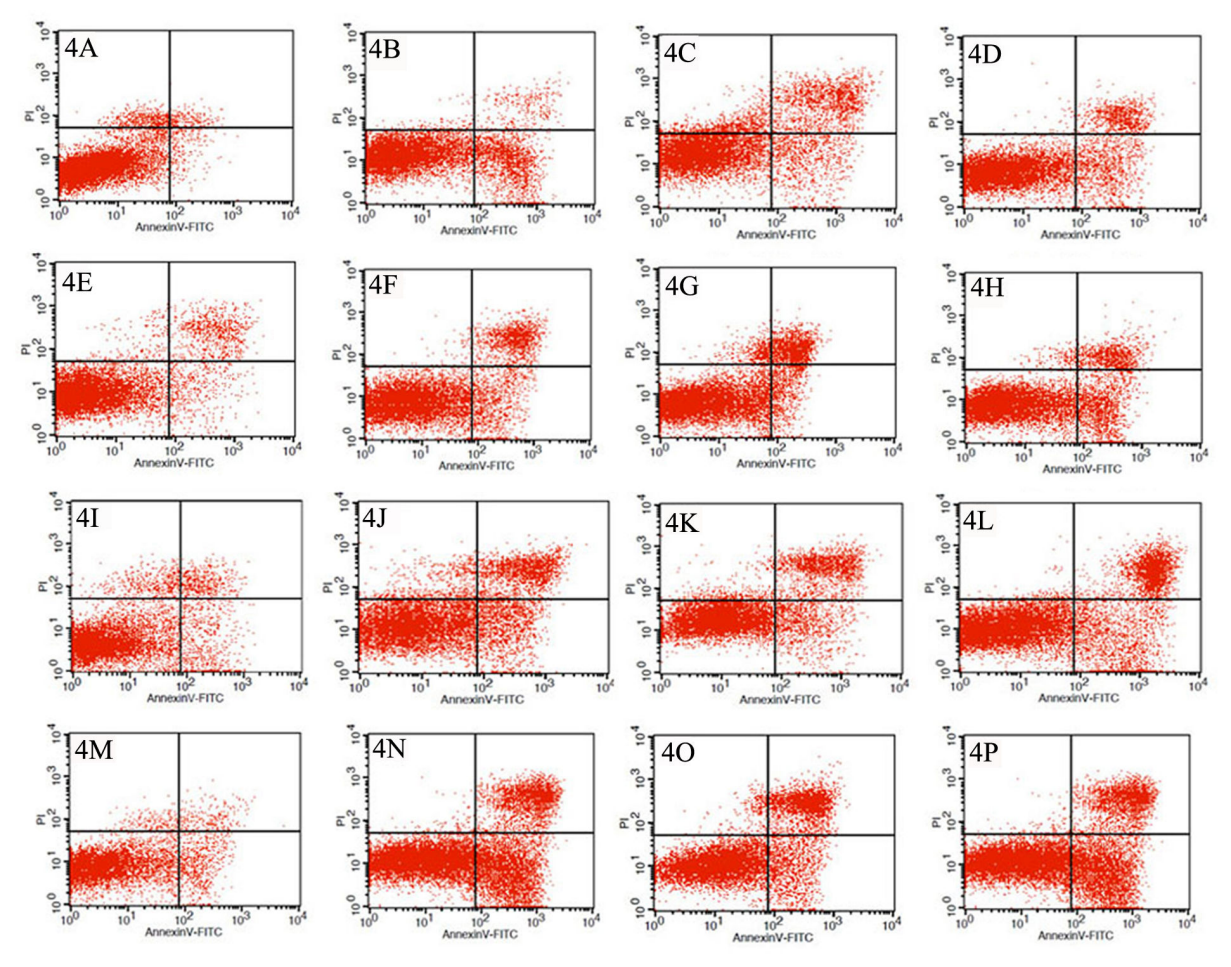
Flow cytometry analysis of hemocytes indicating the percentages of apoptotic cells. Hemocytes were stained with Annexin-V/PI. The lower left (LL) Annexin-V_/PI_ quadrant represents normal healthy cells. The lower right (LR) Annexin-V +/PI_ and upper right (UR) Annexin-V +/PI+ quadrants represent early and late apoptotic cells, respectively. The upper left (UL) Annexin-V_/PI+ quadrant represents necrotic cells. 4A-4D: The results detected at 6 h post-treatment (4A). Control group, (4B) 5.5 µg/mL SMC treatment group, (4C) 55 µg/mL SMC treatment group, and (4D) 550 µg/mL SMC treatment group. 4E-4H: The results detected at 12 h post-treatment (4E). Control group, (4F) 5.5 µg/mL SMC treatment group, (4G) 55 µg/mL SMC treatment group, and (4H) 550 µg/mL SMC treatment group. 4I-4L: The results detected at 24 h post-treatment (4I). Control group, (4J) 5.5 µg/mL SMC treatment group, (4K) 55 µg/mL SMC treatment group, and (4L) 550 µg/mL SMC treatment group;. 4M-4P: The results detected at 36 h post-treatment (4M). Control group, (4N) 5.5 µg/mL SMC treatment group, (4O) 55 µg/mL SMC treatment group, and (4P) 550 µg/mL SMC treatment group.

At 6, 12, 24, and 36 h post-treatment with 5.5, 55, or 550 µg/mL SMC solution, the hemocytes were detected using FCM. The hemocytes were divided into four groups: normal healthy cells, early apoptotic cells, late apoptotic cells, and necrotic cells. The results were analyzed in flow-cytometric data figures with four quadrants: the lower left quadrant (LL, annexin-V-/PI-) corresponds to the normal healthy cells, the lower right quadrant (LR, annexin-V+/PI-), the early apoptotic cells, the upper right quadrant (UR, annexin-V+/PI+), the late apoptotic cells, and the upper left quadrant (UL, annexin-V-/PI+), the necrotic cells (Figure 4).

At 6 h post-treatment, in the untreated control groups, there were 94.31% normal healthy cells, 1.21% early apoptotic cells, 1.14% late apoptotic cells, and 3.34% necrotic cells (Figure 4A, Table 1). In contrast, in the group treated with 5.5 µg/mL SMC solution, the percentages of the four classes of cells were 85.05%, 11.93%, 2.5%, and 0.51%, respectively (Figure 4B, Table 1). In the group treated with 55 µg/mL SMC solution, the percentages of the four classes of cells were 83.6%, 13.1%, 2.91%, and 0.39%, respectively (Figure 4C, Table 1). Finally, in the group treated with 550 µg/mL SMC solution, the percentages of the four classes of cells were 80.50%, 18.42%, 0.53%, and 0.55%, respectively (Figure 4D, Table 1). These results indicated that the observed cellular apoptosis in the hemocytes was caused by the secondary metabolites, and that the apoptotic cells were mostly in early apoptosis at 6 h post-treatment.

**Table 1:** Percentages of apoptotic cells in the flow cytometry figure.

Figure NO	%Gated			
	UL(Necrotic cells)	UR(Late apoptotic cells)	LL(Normal healthy cells)	LR(Early apoptotic cells)
3A(6h-control)	3.34	1.14	94.31	1.21
3B(6h-5.5 µg/mL)	0.51	2.50	85.05	11.93
3C(6h-55 µg/mL)	0.39	2.91	83.80	13.10
3D(6h-550 µg/mL)	0.55	0.53	80.50	18.42
3E(12h-control)	0.43	1.68	94.25	3.64
3F(12h-5.5 µg/mL)	3.71	14.79	74.48	7.02
3G(12h-55 µg/mL)	0.94	10.95	77.72	10.39
3H(12h-550 µg/mL)	2.88	7.27	75.97	13.88
3I(24h-control)	0.10	1.69	90.52	7.69
3J(24h-5.5 µg/mL)	2.00	9.71	74.44	13.84
3K(24h-55 µg/mL)	0.40	15.12	63.06	21.42
3L(24h-550 µg/mL)	2.20	12.92	67.98	16.90
3M(36h-control)	0.89	1.10	90.48	7.53
3N(36h-5.5 µg/mL)	0.56	12.26	77.85	9.33
3O(36h-55 µg/mL)	1.24	12.15	76.27	10.34
3P(36h-550 µg/mL)	0.49	11.89	73.72	13.90

At 12 h post-treatment, in the untreated control groups, there were 94.25% normal healthy cells, 3.64% early apoptotic cells, 1.68% late apoptotic cells, and 0.43% necrotic cells (Figure 4E, Table 1). In the group treated with 5.5 µg/mL SMC solution, the percentages of the four classes of cells were 74.48%, 7.02%, 14.79%, and 3.71%, respectively (Figure 4F, Table 1). In the group treated with 55 µg/mL SMC solution, the percentages of the four classes cells were 77.72%, 10.39%, 10.95%, and 0.94%, respectively (Figure 4G, Table 1). Finally, in the group treated with 550 µg/mL SMC solution, the percentages of the four classes of cells were 75.97%, 13.88%, 7.27%, and 2.88%, respectively (Figure 4H, Table 1). These results indicated that more late-apoptotic cells were characteristic of this period.

At 24 h post-treatment, in the untreated control groups, there were 90.52%, 7.69%, 1.69%, and 0.1% normal healthy, early apoptotic, late apoptotic, and necrotic cells, respectively (Figure 4I, Table 1). In the groups treated with the SMC solution (5.5-550 µg/mL), the percentages of the four classes of cells were 74.44%, 63.06%, and 67.98%; 13.84%, 21.42%, and 16.9%; 9.71%, 15.12%, and 12.92%; and 2%, 0.4%, and 2.2%, respectively (Figure 4J–4L, Table 1). It was observed that both early and late apoptosis of hemocytes increased in this period.

At 36 h post-treatment, in the untreated control groups, there were 90.48% normal healthy cells, 7.53% early apoptotic cells, 1.1% late apoptotic cells, and 0.89% necrotic cells (Figure 4M, Table 1). In contrast, in the SMC-treated groups (5.5-550 µg/mL), the percentages of the four classes of cells were 77.85%, 76.27%, and 73.72%; 9.33%, 10.34%, and 13.90%; 12.26%, 12.15%, and 11.89%; and 0.56%, 1.24%, and 0.49%, respectively (Figure 4N–4P, Table 1). It was observed that the proportions of the early and late apoptotic cells were similar to each other in this period.

The statistical analysis showed that the apoptosis rates of the hemocytes were correlated with the action time and concentration of the metabolite. The highest apoptosis rate was 36.54 ± 4.37%, which appeared at 24 h post-treatment in the group treated with 55 µg/mL. Analyzing the data from the viewpoint of SMC action times, the percentages of apoptotic cells at 24 h were significantly higher than those at the other three periods. While analyzing the data in regards to the effect of the SMC concentration, the percentages of apoptotic cells in the groups treated with 550 µg/mL SMC were significantly higher than those in the groups treated with 5.5 and 55 µg/mL (Table 2).

**Table 2:** Statistical analysis of the percentages (mean ± SE) of apoptotic hemocytes after treatment with the SMC of fungi.

Concentration(μg/mL)		Treated time (h)				Univariate (Concentration)			
		6	12	24	36	Subset	df	F	P
Control		2.34 ± 0.02	8.16 ± 4.05	10.395 ± 1.44	10.08 ± 2.05	c	3.00	68.22	0.00
5.5		16.28 ± 2.17	17.33 ± 7.08	26.82 ± 4.62	17.22 ± 6.18	b			
55		15.71 ± 0.95	20.18 ± 1.65	33.45 ± 4.37	19.40 ± 6.64	ab			
550		18.31 ± 0.91	23.57 ± 3.42	29.17 ± 4.37	24.88 ± 1.29	a			
Univariate (Time)	Subset	c	b	a	b	/			
	df	3.00				/			
	F	28.91				/			
	p	0.00				/			

The different letters in the same line (or column) show significantly different between different treated time (or concentration), P≤0.05.

## Discussion

Insect hemocytes are a complex of cell types that circulate in the hemolymph and play an important immune function in the defense system against invading organisms. Hemocytes are able to recognize foreign bodies in the hemolymph and phagocytose them. Plasmatocytes and granulocytes are the major cell types involved in this phagocytosis [17]. Apoptosis is a form of cell death characterized by cell shrinkage, chromatin condensation and marginalization, and plasma membrane disruption [18]. Zibaee et al. [19] reported that the pathogenic mechanism of the entomogenous fungus *Beauveria bassiana* (Bals.) Vuill (Ascomycota: Hypocreales) is to disable the immune function of the *Eurygasterintegriceps* Puton (Hemiptera: Scutelleridae) pest through secondary metabolites. However, studies on the effects of fungal secondary metabolites on insect hemocyte apoptosis are scarce, in spite of the fact that some fungal metabolite toxins have been known for years [20,21].

Our study showed that the secondary metabolites of *B* *. brongniartii* were able to induce hemocyte apoptosis in *D* *. tabulaeformis* larvae. By using fluorescence microscopy, we clearly observed that the early apoptotic hemocytes radiated a non-uniform kelly fluorescence, the late apoptotic cells radiated a non-uniform orange fluorescence, and the normal healthy hemocytes radiated a uniform green fluorescence. The AO/PI double staining technique has been used by other researchers to test apoptosis of human cells and their results were similar to ours [15,16]. Wahab et al. [15] reported that the apoptogenic effects of zerumbone on cancer cells of the human cervix (HeLa) were studied using fluorescence microscopy (AO/PI double staining). The results showed that the HeLa cells in early apoptosis clearly emitted a bright-green fluorescence, with fragmented DNA. In addition, cells in late apoptosis emitted a reddish-orange fluorescence, with apoptotic bodies. In contrast, the normal cells emitted a green fluorescence with an intact nuclear structure.

Da-Silveira et al. [22] used the TEM to observe the ultra-structural symptoms of insect hemocyte apoptosis in their study of an *Anticarsia* *gemmatalis* *multiple nucleopolyhedrovirus* (AgMNPV) mutant, vApAg, inducing hemocyte apoptosis in *Anticarsia* *gemmatalis* Hubner (Lepidoptera, Noctuidae) (UFL-AG-286). However, we observed nuclear hypertrophy, nuclear envelope folding, chromatin concentration, fibrillar aggregation in the cytoplasm, and chondriosome changes during hemocyte apoptosis in *D* *. tabulaeformis* larvae that were treated with the secondary metabolites of *B* *. brongniartii* .

Although flow cytometry has been widely used for the rapid analysis of a mass of cells undergoing apoptosis in recent years, this technology is still in a tentative stage in the study of insect cell apoptosis. Using flow cytometry, Xia et al. [23] reported the induction of hemocyte apoptosis in *Spodoptera* *litura* Fab. (Lepidoptera: Noctuidae) larvae by the heavy metal zinc. They used the dual dye Hoechst 33258/PI in flow cytometry and distinguished cellular apoptosis from necrosis. We employed the dual dye Annexin-V FITC/PI and acquired more accurate information about hemocyte apoptosis in *D* *. tabulaeformis* larvae as shown in Figure 4. Using flow cytometry, the numbers and percentages of the normal, early apoptotic, later apoptotic, and necrotic hemocytes were measured. We found that under the action of the secondary metabolites of *B* *. brongniartii* , early apoptosis of the hemocytes occurred mainly at 6 h post-treatment and late apoptosis occurred mostly at 12 h post-treatment.

The response of the hemocytes against the invading substance is a rapid-acting immune response that represents a primary means of limiting microbial infection in some species of arthropods. For example, Oliver et al. examined this at 1 h post-injection and found the extent of phagocytosis among a wide taxonomic range of arthropod species, including a decapod crustacean ( *Litopenaeusvannamei* ), three ixodid tick species ( *Amblyomma* *americanum* *,* *Dermacentor* *variabilis* *, and* *Ixodes* *scapularis* ), a mosquito species (*Aedes aegypti*), and a larval moth (*Manduca sexta*) [24]. Meanwhile, apoptosis is an energy-requiring, programmed physiological process that eliminates superfluous, altered, or malignant cells [25]. In some species of Insecta, hemocyte apoptosis occurs slower after foreign bodies invade the hemocoel of the host. Hemocytes of the *S* *. litura* larvae undergoing apoptosis were detected at 2, 12, 24, 48, and 72 h after the larvae were injected with *M* *. bicoloratus* ovarian calyx fluid [25]. In our study, hemocyte apoptosis was detected from 6 to 36 h, but the early apoptosis of hemocytes occurred mainly at 6 h post-treatment and late apoptosis occurred mostly at 12 h post-treatment. These differences in cellar apoptosis indicate apoptosis is a genetically controlled process.

The total number of hemocytes declines when insects defend against the invading substance [13,19]. Zibaee et al. [19] reported that the total hemocyte counts of *E* *. integriceps* declined after treatment with secondary metabolites of *B. bassiana*. Apoptosis may play an important role in the reduction of the total number of hemocytes. In our study, a high dose (550 µg/mL) SMC solution resulted in a high percentage of early apoptotic cells and a low percentage of late apoptotic cells (Table 1). This phenomenon could be caused by high dose accelerating disintegration of the late apoptotic cells and phagocytosis of the cell fragments by neighboring cells.

In general, the secondary metabolites of *B* *. brongniartii* were able to attack the hemocytes of *D* *. tabulaeformis* larvae and induce cellular apoptosis. Hence, the secondary metabolites were able to inhibit the immune activity of the hemocytes. These results provided new evidence that insect hemocytes are among the targets of the secondary metabolites of the mycopathogens. When infecting insects, the fungus can metabolize certain secondary metabolites as a weapon to cause hemocyte apoptosis and necrosis and thus destroy the immune function of the host hemocytes.

## Materials and Methods

### Ethics Statement

The collection of *D* *. tabulaeformis* was permitted by Forestry Disease and Pest Control Station of Lingqiu County, Shanxi Province, China.

### Insect

The larvae of *D* *. tabulaeformis* were collected from the pine forest in Lingqiu (114°22′E 39°44′N) in the Shanxi province in Northern China. The larvae were reared with fresh pine needles of *Pinus* *tabulaeformis* Carr (Coniferopsida: Pinaceae). in a rearing room at 27 ± 1 °C, 75 ± 10% relative humidity (RH), and a 15:9 (L: D) photoperiod. After two generations, the healthy larvae in the 4^th^ instar stage were used for the experiments.

### Entomopathogenic fungus

The entomopathogenic fungus *B* *. brongniartii* strain 2382 was employed in these experiments. This strain was originally isolated by our laboratory from the naturally infected larvae of *D* *. tabulaeformis* , which were collected from a pine forest in Chengde (117°51′E 40°57′N) in the Hebei Province in China and synchronously stored in the China General Microbiological Culture Collection [26]. Before the experiment, the strain was cultured on potato-dextrose-agar medium for 15 days at 25 ± 1 °C and 75 ± 10% RH to harvest conidia.

### Fungal liquid culture

After the harvest, conidia were prepared as a suspension with a concentration of 1 × 10^8^ spores/mL. A 1 mL aliquot of the conidial suspension was used to inoculate 100 mL of liquid medium [27] that was prepared with 10 g/L peptone, 10 g/L yeast extract, and 10 g/L glucose, in a 250 mL conical flask. The fungal culture was kept in an incubator (Thermo Scientific MAXQ 5000, Rockford, USA) at a constant temperature of 25 ± 1 °C and shaken at a constant rate of 165 rpm for 7 days.

### Extraction of the fungal secondary metabolite

After culturing, crude extracts of the cultured broth were obtained following the method reported by Hu [27]. The fermentation broth was centrifuged (Eppendorf Centrifuge 58042, Hamburg, Germany) at 10,000 × g for 15 min and then concentrated at 50 °C. The concentrated broth was then precipitated with alcohol for 24 h and the final concentration was 70% [v/v] (Kermel Chemical Reagent Co., Tianjin, China). After centrifugation (Eppendorf Centrifuge 58042) at 5,000 × g for 20 min, the supernatant was sequentially extracted with ethyl acetate (Kermel) at a 1:2 ratio. Finally, an orange-red powder was obtained after drying at 40 °C. This orange-red powder was the SMC of the fungus and was used in the bioassays.

### Bioassays

In the bioassays, the SMC of the fungus was prepared in solutions of three concentrations, 5.5, 55, and 550 µg/mL, and the lack of the SMC was the control. These concentrations were picked based on the formula LC_50_=280 µg/mL, from our previous experiment [28]. The samples of test larvae were inoculated with 1 µL of the SMC solutions by injection into their bodies using a microinjector (Angle, Shanghai, China). Sixty samples of larvae were separately inoculated in each experimental group and an additional 60 larval samples were synchronously treated with 0.5% DMSO (Sigma-Aldrich, St. Louis, USA) as the control. The experiments were conducted twice with three replicates each. The inoculated insects were transferred to rearing chambers (Boxun SPX-2051-C, Shanghai, China) and cultured for 6 days at 25 ± 1 °C, with 75 ± 10% RH and a photoperiod of 15: 9 h (L: D).

### Collection of hemocytes

Five insects in each experimental group were randomly sampled at 6, 12, 24, and 36 h post-treatment. The insect samples were cut at the base of the 3rd pair of abdominal legs with microscopic scissors and the hemolymph was collected. Three replicates were conducted. Two hundred microliters of hemolymph was introduced into a 1.5 mL Eppendorf tube containing 300 µL of an ice-cold anti-coagulant solution (98 mM NaOH, 186 mM NaCl, 17 mM EDTA, 41 mM citric acid, pH 4.5) [29]. Hemocytes were separated from the hemolymph solution by centrifugation (Eppendorf Centrifuge 58042) at 1,500 × g for 10 min at 4 °C, washed, and resuspended in anti-coagulant solution [30].

### Fluorescence microscopy

The samples of hemocytes collected from the larvae at 6 h and 12 h post-treatment were used for cell observation by fluorescence microscopy. We used AO/PI to double stain the cells because the cellular fluorescence was more distinct and was maintained for a longer time using this method. One hundred microliters of the cell suspensions was stained with 5 µL AO/PI (100 mg/mL) (Sigma-Aldrich), and the stained cells were observed and photographed at 365 nm under a fluorescence microscope (Olympus BX51, Tokyo, Japan). The microscope was equipped with a super high-pressure mercury lamp and connected to a DS cooled camera head (DS-5Mc) regulated by ACT-2U software (Nikon Corporation, Tokyo, Japan).

### Transmission electron microscopy

At 12 h and 24 h post-treatment, hemocyte samples from the test group treated with 55 µg/mL of the SMC solution were immersed in 4% (v/v) glutaraldehyde (Sigma-Aldrich) in a 0.2 M phosphate buffer at a pH of 7.2 and a temperature of 4 °C for 48 h, post-fixed in 1% (v/v) osmium tetroxide (Sigma) in a phosphate buffer for 3 h at 4 °C, dehydrated with a series of ethanol (Kermel) solutions (10%-100%), and embedded in Epon 812 (Fluka, USA). Ultrathin sections (0.08 µm), cut using a Reichert Jung ultramicrotome, were collected on copper grids and counterstained with uranyl acetate and lead citrate. The ultrathin sections were examined using a JEM-1200EX transmission electron microscope (JEOL Ltd., Mitaka, Japan) with an accelerating voltage of 80 kV. Micrographs were taken on a Lucky TEM negative film (Lucky company Inc., Baoding, China) using a microscope camera equipped with the JEM-1200EX TEM. The films were developed and scanned using an N-TEK Nuscan 700 scanner (Microtek Ltd., Shanghai, China).

### Flow cytometry

The collected hemocytes were washed twice with PBS (0.2 M, pH7.2) and resuspended in 500 µL Annexin-V Binding Buffer. Next, 5 µL of Annexin-V FITC (BD Ltd, Franklin Lakes, USA) and 10 µL of Propidium Iodide Buffer (BD) were added to each tube and the samples were incubated at room temperature for 15 min in the dark. Finally, within 1 h of staining, the cells were analyzed by FCM. A total of 10,000 cells were counted in each treatment and the experiments were replicated three times.

### Statistical analysis

The data were compared using the Tukey method in Univariate of the General Linear Model when significant differences were found at P≤0.05 (SPSS 16.0). The differences between samplings were considered statistically significant at a probability of less than 5%.

## References

[1] Roberts DW, St Leger RJ (2004) *Metarhizium* spp., cosmopolitan insect-pathogenic fungi: mycological aspects. Adv Appl Microbiol 54: 1–70. doi:10.1016/S0065-2164(04)54001-7. PubMed: 15251275. 15251275 10.1016/S0065-2164(04)54001-7

[2] Wang JJ, Cheng WX, Ding W, Zhao ZM (2004) Foraging behavior and prey interactions by a guild of predators on various lifestages of Bemisia tabaci. J Insect Sci 4: 1–5. PubMed: 15861217. 15861217 10.1673/031.004.3101 PMC455675

[3] Thomas MB, Read AF (2007) Can fungal biopesticides control malaria? Nat Rev Microbiol 5: 377–383. doi:10.1038/nrmicro1638. PubMed: 17426726. 17426726 10.1038/nrmicro1638

[4] Goettel MS, Duke GM, Goerzen DW (1997) Pathogenicity of *Ascosphaera larvis* to larvae of the alfalfa leafcutting bee, *Megachile rotundata* . Can Entomol 129: 1059–1065. doi:10.4039/Ent1291059-6.

[5] Hu QB, Ren SX, Wu JH, Chang JM, Musa PD (2006) Investigation of destruxin A and B from 80 *Metarhizium* strains in China, and the optimization of cultural conditions for the strain MaQ10. Toxicon 48: 491–498. doi:10.1016/j.toxicon.2006.06.018. PubMed: 16956639. 16956639 10.1016/j.toxicon.2006.06.018

[6] Ortiz-Urquiza A, Vergara-Ortiz A, Santiago-Alvarez C, Quesada-Moraga E (2010) Insecticidal and sublethal reproductive effects of *Metarhizium anisopliae* culture supernatant protein extract on the Mediterranean fruit fly. J Appl Entomol 134: 581–591.

[7] Hajek AE, St-Leger RJ (1994) Interaction between fungal pathogens and insect hosts. Annu Rev Entomol 39: 293–332. doi:10.1146/annurev.en.39.010194.001453.

[8] Hoffmann JA, Kafatos FC, Janeway CA-Jr, Ezekowitz RA (1999) Phylogenetic perspectives in innate immunity. Science 284: 1313–1317. doi:10.1126/science.284.5418.1313. PubMed: 10334979. 10334979 10.1126/science.284.5418.1313

[9] Carton Y, Nappi AJ (1997) Drosophila cellular immunity against parasitoids. Parasitol Today 13: 218–227. doi:10.1016/S0169-4758(97)01058-2. PubMed: 15275074. 15275074 10.1016/s0169-4758(97)01058-2

[10] Jiravanichpaisal P, Lee BL, Söderhäll K (2006) Cell-mediated immunity in arthropods: Hematopoiesis, coagulation, melanization and opsonization. Immunobiology 211: 213–236. doi:10.1016/j.imbio.2005.10.015. PubMed: 16697916. 16697916 10.1016/j.imbio.2005.10.015

[11] Lavine MD, Strand MR (2002) Insect hemocytes and their role in immunity. Insect Biochem Mol Biol 32: 1295–1309. doi:10.1016/S0965-1748(02)00092-9. PubMed: 12225920. 12225920 10.1016/s0965-1748(02)00092-9

[12] Schmidt O, Theopold U, Strand M (2001) Innate immunity and its evasion and suppression by *Hymenopteran endoparasitoids* . BioEssays 23: 344–351. doi:10.1002/bies.1049. PubMed: 11268040. 11268040 10.1002/bies.1049

[13] Bandani AR (2005) Effects of *Tolypocladium cylindrosporum* and its secondary metabolites, efrapeptins, on the immune system of *Galleria mellonella* larvae. Biocon Sci Technol 15: 67–79. doi:10.1080/09583150400015961.

[14] Fan JH, Xie YP, Xue JL, Li BZ (2008) Isolation and identification of toxins inhibiting *Dentrolimus tabulaeformis* from an antagonistic strain of *Beauveria* . Acta Microbiol Sin 48: 596–601. 18652290

[15] Abdel Wahab SI, Abdul AB, Alzubairi AS, Mohamed Elhassan M, Mohan S (2009) In vitro ultramorphological assessment of apoptosis induced by zerumbone on (HeLa). J Biomed Biotechnol, 2009: 2009: 769568. PubMed: 19343171 10.1155/2009/769568 PMC2661117 19343171

[16] Tajudin TJSA, Mat N, Siti-Aishah AB, Yusran AAM, Alwi A et al. (2012) Cytotoxicity, antiproliferative effects, and apoptosis induction of methanolic extract of *Cynometra cauliflora* Linn. whole fruit on human promyelocytic leukemia HL-60 cells. Evidence-Based Compl Altern Med: 2012: 127373 10.1155/2012/127373 PMC3513972 23227094

[17] Klowden MJ (2007) Physiological systems in insects, second edition. Beijing: Science Press. 377pp.

[18] Yedjou CG, Milner JN, Howard CB, Tchounwou PB (2010) Basic apoptotic mechanisms of lead toxicity in human leukemia (HL-60) cells. Int J Environ Res Public Health 7: 2008–2017. doi:10.3390/ijerph7052008. PubMed: 20623007. 20623007 10.3390/ijerph7052008 PMC2898032

[19] Zibaee A, Bandani AR, Talaei-Hassanlouei R, Malagoli D (2011) Cellular immune reactions of the sunn pest, *Eurygaster integriceps*, to the entomopathogenic fungus, *Beauveria bassiana* and its secondary metabolites. J Insect Sci 11: 138 PubMed: 22233481. 22233481 10.1673/031.011.13801 PMC3391913

[20] Meng X, Xu X, Hu J, Jin F, Hu Q et al. (2011) Toxicity and differential protein analysis following destruxin A treatment of *Spodoptera litura* (Lepidoptera: Noctuidae) SL-1 cells. Toxicon 58: 327–335. doi:10.1016/j.toxicon.2011.06.002. PubMed: 21718714. 21718714 10.1016/j.toxicon.2011.06.002

[21] Zibaee A, Bandani AR, Tork M (2009) Effect of the entomopathogenic fungus, *Beauveria bassiana*, and its secondary metabolite on detoxifying enzyme activities and acetylcholinesterase (AChE) of the Sunn pest, *Eurygaster integriceps* (Heteroptera: Scutellaridae). Biocon Sci Technol 19: 485–498. doi:10.1080/09583150902847127.

[22] da Silveira EB, Cordeiro BA, Ribeiro BM, de Castro ME, Soares EF et al. (2007) An Anticarsia gemmatalis multiple nucleopolyhedrovirus mutant, vApAg, induces hemocytes apoptosis in vivo and displays reduced infectivity in larvae of Anticarsia gemmatalis (Hübner) (Lepidoptera: Noctuidae). Virus Res 130: 182–192. doi:10.1016/j.virusres.2007.06.010. PubMed: 17643541. 17643541 10.1016/j.virusres.2007.06.010

[23] Xia Q, Sun H, Hu X, Shu Y, Gu D et al. (2005) Apoptosis of *SpoJoptera litura* larval hemocytes induced by heavy metal zinc. Chin Sci Bull 50: 2856–2860. doi:10.1007/BF02899656.

[24] Oliver JD, Dusty Loy J, Parikh G, Bartholomay L (2011) Comparative analysis of hemocyte phagocytosis between six species of arthropods as measured by flow cytometry. J Invertebr Pathol 108: 126–130. doi:10.1016/j.jip.2011.07.004. PubMed: 21843526. 21843526 10.1016/j.jip.2011.07.004

[25] Luo K, Pang Y (2006) *Spodoptera litura* multicapsid nucleopolyhedrovirus inhibits *Microplitis bicoloratus* polydnavirus-induced host granulocytes apoptosis. J Insect Physiol 52: 795–806. doi:10.1016/j.jinsphys.2006.04.007. PubMed: 16764883. 16764883 10.1016/j.jinsphys.2006.04.007

[26] Fan JH, Xie YP, Xue JL, Li BZ (2007) Isolation, identification and biological characteristics of a Strain of the entomopathogenic fungus, *Beauveria tenella* parasitizing *Dentrolimus tabulaeformis* . J Shanxi Univ (Nat Sci) 30: 107－110.

[27] Hu FL, Ding XJ, Yang C, Li ZZ, Fan MZ (2006) Isolation and identification of monoamine oxidase inhibitor from fermentation broth of a strain of Beauveria. Mycosystema 25: 273–277.

[28] Fan JH, Xie YP, Xue JL, Liu R (2013) The effect of *Beauveria brongniartii* and its secondary metabolites on the detoxification enzymes of the pine caterpillar, *Dendrolimus tabulaeformis* . J Insect Sci 13: 44 Available: http://www.insectscience.org/13.44. Accessed: 21 May 2013. 23909949 10.1673/031.013.4401 PMC3740923

[29] Mead GP, Ratcliffe NA, Renwarntz LR (1986) The separation of insect hemocyte types on percoll gradients; methodology and problems. J Insect Physiol 32: 167–177. doi:10.1016/0022-1910(86)90137-X.

[30] Masahiro S, Ken M, Toshiharu T (2009) Effects of the virus-like particles of a braconid endoparasitoid, *Meteorus pulchricornis*, on hemocytes and hematopoietic organs of its noctuid host, *Pseudaletia separate* . Appl Entomol Zool 44: 115–125. doi:10.1303/aez.2009.115.

